# Asthma

**Published:** 1870-12

**Authors:** 


### ASTHMA.

A martyr to this terrible disease, which seldom kills any one dead, yet makes its victim die a thousand deaths, will be glad to know of any alleviant. A simple change of air from moist to dry, from the city to the prairie, from plain to mountain, or the reverse, has been known to be beneficial. Persons ascending the mountain heights of Cornwall, on the Hudson, near West Point, have had striking relief within a few hours. A gentleman once found instant relief by being closeted with a pole-cat; he was so delighted that he obtained the musk-bag, put it in a bottle of whiskey, drank it daily from time to time, and was permanently cured. That instantaneous relief was given, is certain—whether the permanent cure was effected by musk-bag tincture, is conjecture.

